# Supplementary material for: Passive mode-locking and terahertz frequency comb generation in resonant-tunneling-diode oscillator
Source: Nat Commun. 2022 Jun 29;13:3740. doi: 10.1038/s41467-022-31071-3 (PMC9243027; doi:10.1038/s41467-022-31071-3)
Supplement: Supplementary file 1 — Supplementary Information [file 41467_2022_31071_MOESM1_ESM.pdf]

Supplementary information

Passive mode-locking and terahertz frequency comb generation in resonant-tunneling-diode oscillator

Tomoki Hiraoka[1]*, Yuta Inose[1]*, Takashi Arikawa[1,2], Hiroshi Ito[3], and Koichiro Tanaka[1]*
(* corresponding author)

[1] *Department of Physics, Graduate School of Science, Kyoto University, Sakyo-ku, Kyoto 606-8502, Japan*
[2] *PRESTO, Japan Science and Technology Agency (JST), 4-1-8 Honcho, Kawaguchi 332-0012, Japan*
[3] *Center for Natural Sciences, Kitasato University, Minami-ku, Sagamihara 252-0373, Japan*

*Corresponding Author:
Tomoki Hiraoka: t.hiraoka1023@gmail.com
Yuta Inose: inose.yuta.t14@kyoto-u.jp
Koichiro Tanaka: kochan@scphys.kyoto-u.ac.jp

Supplementary information

Note 1 Detail of the experimental setup

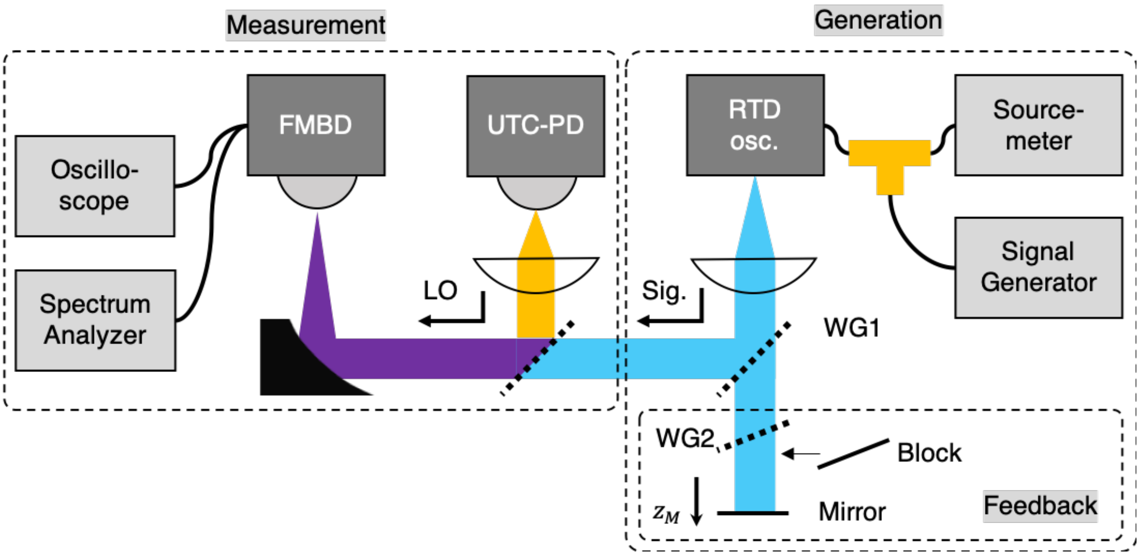

Fig. S1 Detailed experimental setup. Abbreviations are as follows: WG; wire-grid polarizer, Sig.; signal, LO; local oscillator, UTC-PD; uni-traveling-carrier photodiode, FMBD; Fermi-level managed barrier diode.

Figure S1 shows the detailed experimental setup.

Note 2 **Analytical model of feedback effect on the oscillation frequency**

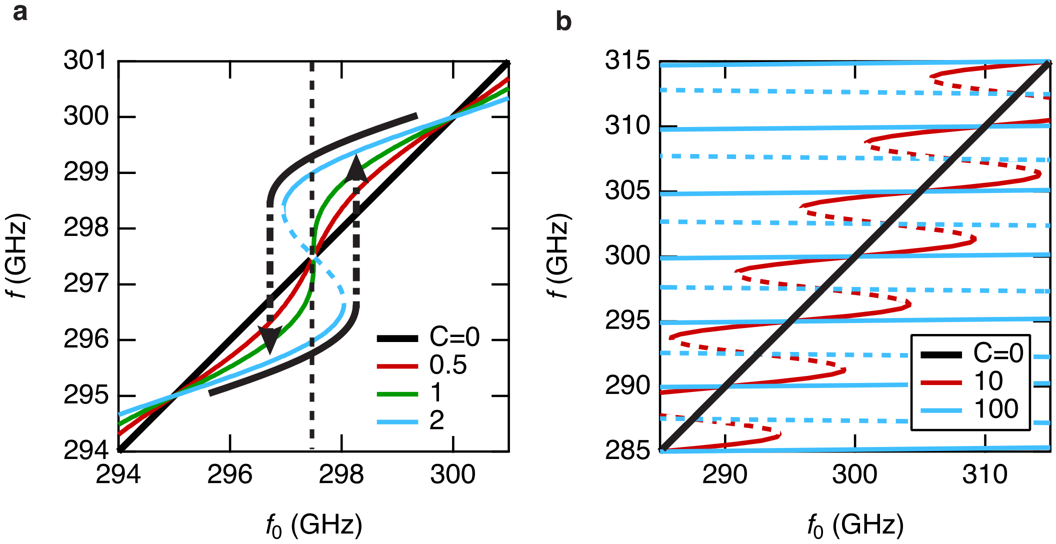

Fig. S2 Relation of oscillation frequency $f = \omega/2\pi$ and the free-running frequency $f_0 = \omega_0/2\pi$ derived from equation (2) for $f_0 = 300$ GHz, $t_d = 2 \times 10^{-10}$ s (corresponds to an optical distance of 30 mm) and various feedback parameters C. The dotted curves with negative slopes are unstable solutions [1]. **a**, $C = 0, 0.5, 1$ and 2. The black arrow shows the frequency change observed when f_0 is swept. **b**, $C = 0, 10$ and 100.

In this note, we show an analytical model to explain the optical feedback effect on the oscillation frequency, i.e., the modification of the frequency-voltage curve, appearances of multiple optical modes, and dependence of the mode spacing to the feedback amplitude.

Here, we use the model derived in the reference [2]. They modeled the RTD oscillator under optical feedback with the following circuit equation:

$$C_{ant}\frac{d^2V}{dt^2} + \left(G_r - G_{rtd} - 2\sqrt{\eta}G_r \exp[-j(2k_0 l + \phi)]\right)\frac{dV}{dt} + \frac{V}{L} = 0. \tag{1}$$

Here, C_{ant} is the capacitance of the resonator, G_r is the radiation conductance, and G_{rtd} is the absolute value of the negative differential conductance. η is the *reflectivity* of the return light, including the coupling efficiency. $2k_0 l$ is the phase delay of the return light, and ϕ is a constant phase shift of the return light. The condition for steady oscillation in equation (1) yields the following equation:

$$\omega t_d \cong \omega_0 t_d - \frac{\sqrt{\eta}\omega_0 t_d}{Q}\sin(\omega t_d + \phi) = \omega_0 t_d - C\sin(\omega t_d + \phi). \tag{2}$$

Here, ω is the oscillation frequency under the influence of the feedback, ω_0 is the free-running oscillation frequency, and Q is the quality factor of the LCR resonator. t_d is the delay time, and

$$C = \sqrt{\eta}\omega_0 t_d/Q \tag{3}$$

is a feedback parameter. Equation (2) represents the change of the oscillation frequency due to optical feedback. Interestingly, the same equation as (2) can be obtained for a semiconductor laser under weak optical feedback [1].

Equation (2) gives the relationship between the oscillation frequency $f = \omega/2\pi$ and the free-running frequency $f_0 = \omega_0/2\pi$. Figure S2 is the numerical plot for various feedback parameters C. This plot corresponds to the frequency-voltage curve because the voltage sweep causes the sweep of the free-running frequency. $C = 0$ is the case of no feedback and $\omega = \omega_0$. Small feedback of $C = 0.5$ makes a slight modulation of ω from ω_0. When the feedback parameter C is unity, ω shows a sudden change around the vertical dashed line. In the case of $C = 2$, ω becomes a multivalued function of ω_0. The curve with a negative slope (dotted line) is an unstable steady-state [1]. Around the multivalued part, sweeping ω_0 causes the frequency jumps and hysteresis of the frequency depending on the sweep direction. It corresponds to a frequency jump and hysteresis observed in the experiment. The frequency difference between the multiple stable states for a certain value of f_0 corresponds to the mode spacing at the corresponding bias voltage. In the weak feedback regime shown in Figure. S2a, the mode spacing is different from the free-spectral range of a Fabry-Perot cavity, i.e., $1/t_d$ (= 5 GHz in Figure. S2). As the feedback amplitude increases more ($C = 10$ and 100), the number of the modes for a certain f_0 increases, and the mode spacing approaches $1/t_d$ (Figure. S2b). The strong-field limit corresponds to the Fabry-Perot cavity.

Note 3 **Temporal waveform in long span**

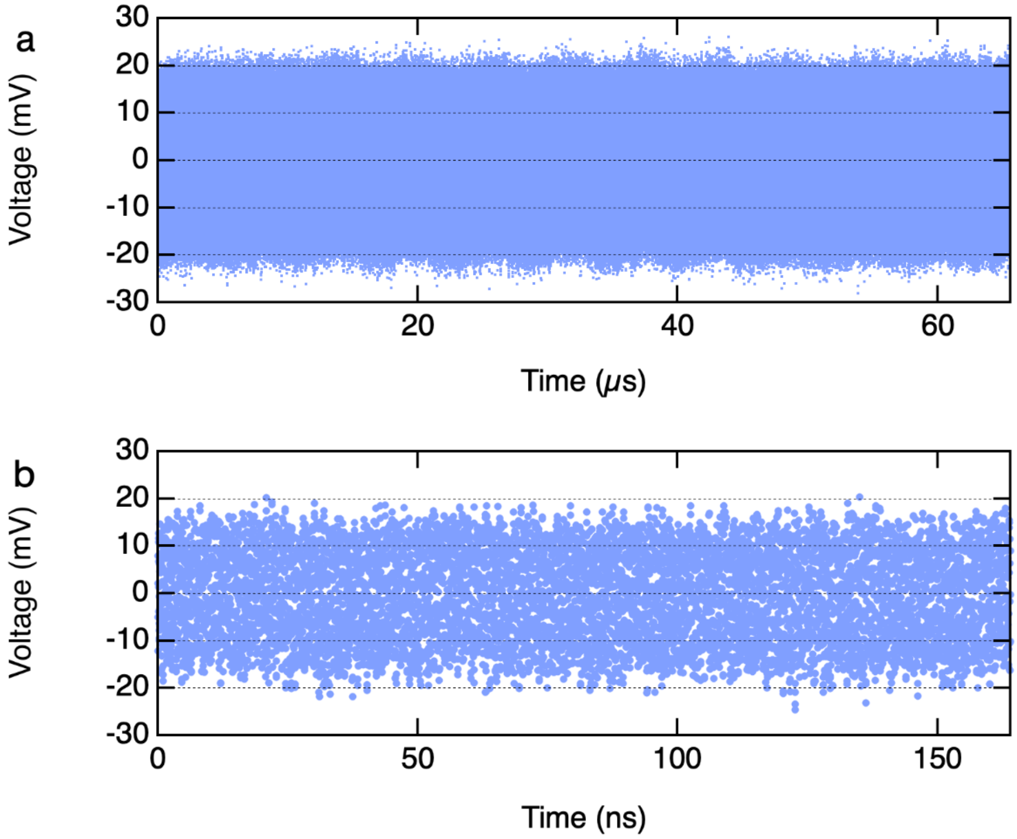

Fig. S3 Measured temporal heterodyne waveform shown in various lengths. Dots in each figure are the data points. **a**, Measured waveform over 65.6 μs. **b**, Measured waveform over 164 ns, which is a single fitting span.

Figure S3 shows the measured temporal waveform plotted for a longer span than that of Figure 2a.

Note 4 Detail of the fitting

(1) Fitting in short spans

The exact value of the time span (164 ns) was determined so that the number of the contained data points is an integer power of 2, for the convenience in the analysis based on Fast Fourier Transform (FFT) algorithm such as the Hilbert transform performed in Supplementary Note 5. The fitting in the divided spans can be explicitly expressed as follows:

$$f_i(t) = \sum_n A_n \sin\left[2\pi\left(f_{0,i}^{\mathrm{RF}} + n f_{\mathrm{rep},i}\right)\left(t - t_{0,i}\right) - \varphi_{n,i}\right]. \quad (4)$$

Here, $f_{0,i}^{\mathrm{RF}}$, $f_{\mathrm{rep},i}$, $t_{0,i}$, and $\varphi_{n,i}$ are the fitting parameter in each short span, corresponding to those in equation (3) in the main text. A_n is derived from the area of the comb lines in the magnified spectra. A constant-coefficient $k = 0.95$ was multiplied to all the amplitudes to correct the difference of the amplitude between the spectrum measurement and the waveform measurement. Since the modes have different frequencies, the relation between the initial phases $\varphi_{n,i}$ depends on the choice of $t_{0,i}$. To express the phase relationship uniquely, we choose the time origin $t_{0,i}$ as the timing when $\varphi_{3,i} = \varphi_{4,i}$ stands, as described in the next section. As a fitting condition, we put a constraint that $\varphi_{3,i} = \varphi_{4,i}$.

(2) Uniqueness of the phase representation

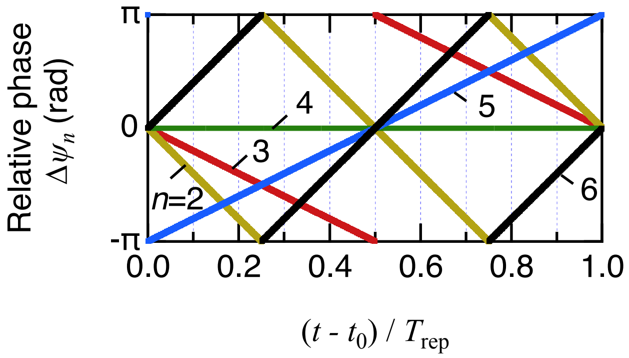

Figure S3 Temporal evolution of the modal phases $\psi_{n,i}$ relative to $\psi_{4,i}$. The initial condition is $(\Delta\varphi_{2,i}, \Delta\varphi_{3,i}, \Delta\varphi_{4,i}, \Delta\varphi_{5,i}, \Delta\varphi_{6,i}) = (0,0,0,\pi,0)$.

Here, we show that the relative initial phase $\Delta\varphi_{n,i} \equiv \varphi_{n,i} - \varphi_{4,i}$ depends on the choice of the time origin $t_{0,i}$, and there is a countless number of the equivalent representations on the phase relationship. We also show how we choose the $t_{0,i}$ in which we can uniquely represent it.

Let us consider equation (4), and the temporal evolution of the modal phases described as

$$\psi_{n,i} = 2\pi\left(f_{0,i}^{\rm RF} + n f_{{\rm rep},i}\right)\left(t - t_{0,i}\right) + \varphi_{n,i}. \tag{5}$$

In the analysis of the mode-locking, we are interested in the relative phase between the modes. If we take the mode with index $n = 4$ as the reference, the relative phase can be defined as

$$\Delta\psi_{n,i} = \psi_{n,i} - \psi_{4,i} = 2\pi(n-4) f_{{\rm rep},i}\left(t - t_{0,i}\right) + \Delta\varphi_{n,i}. \tag{6}$$

Figure S3 shows the temporal evolution of the relative phases $\Delta\psi_{n,i}$ when the initial condition is $(\Delta\varphi_2, \Delta\varphi_3, \Delta\varphi_4, \Delta\varphi_5, \Delta\varphi_6) = (0, 0, 0, \pi, 0)$. The bottom axis shows the time originated at $t_{0,i}$ and normalized with the period $T_{\rm rep} = 1/f_{\rm rep}$. The modes have different frequencies for each other, and the evolution of the relative phase $\Delta\psi_{n,i}$ is dependent on the modal index n.

We note that there is an arbitrariness in the choice of the time origin $t_{0,i}$. For example, we can choose the time $t = t_{0,i} + 0.5\, T_{\rm rep}$ as a new time origin $t_{0,i}^*$. In that case, the relative phase can be written as

$$\Delta\psi_{n,i} = 2\pi(n-4) f_{{\rm rep},i}\left(t - t_{0,i}^*\right) + \Delta\varphi_{n,i}^* \tag{7}$$

with the initial relative phases of $(\Delta\varphi_{2,i}^*, \Delta\varphi_{3,i}^*, \Delta\varphi_{4,i}^*, \Delta\varphi_{5,i}^*, \Delta\varphi_{6,i}^*) = (0, \pi, 0,0,0)$. Hence, the phase relationship has countless number of the equivalent representations.

To represent the phase relationship uniquely, we choose the time where $\varphi_{3,i} = \varphi_{4,i}$ stands as the origin. This condition identifies the time origin in the period of $T_{\rm rep}$ uniquely because it is only one time that the phases of the adjacent modes are equal.

We note that $t_{0,i}$ is necessary because noise induces unexpected timing shifts on the oscillator.

Note 5 **Envelope and instantaneous frequency of passive mode-locked state**

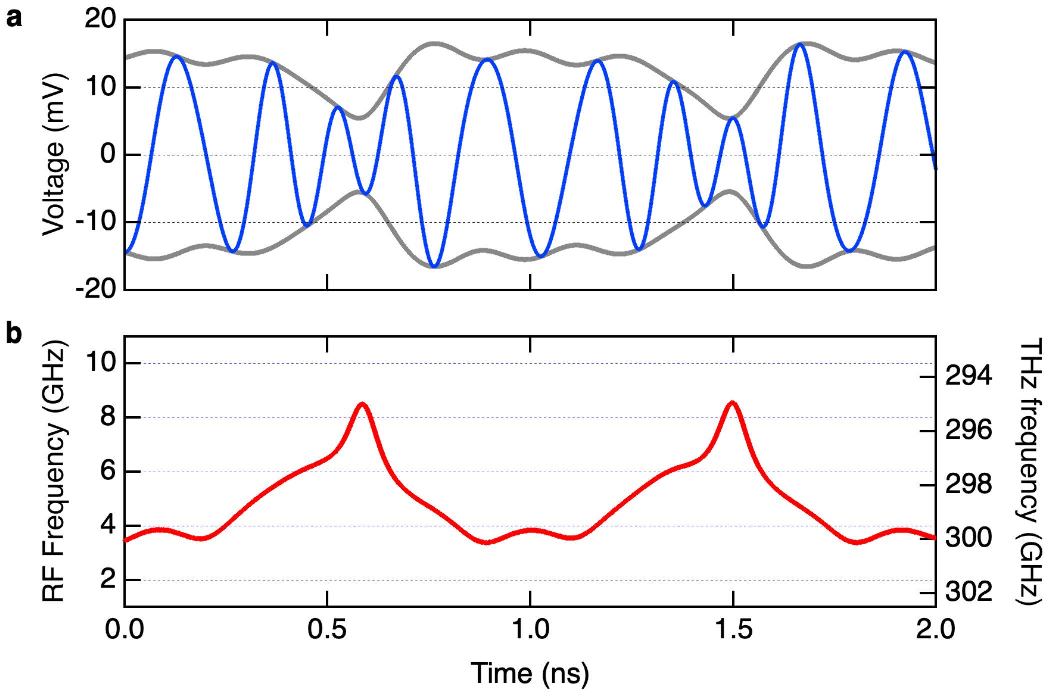

Figure S5 Envelope and instantaneous frequency of the fitted heterodyne waveform, derived from the Hilbert transform. **a**, The typical fitting curve (blue trace) and the envelope (gray trace). The typical fitting curve was obtained by averaging the fitting parameters $\varphi_{n,i}$, $f_{\mathrm{rep},i}$, and $f_{0,i}$ over all the fitting spans. **b**, The instantaneous frequency of the heterodyne waveform and the corresponding terahertz frequency.

Figure S5a shows a typical heterodyne waveform (blue trace) and its envelope (gray trace). Here, we do not discuss amplitude modulation in detail due to the possible inaccuracy of the amplitude measurement.

Figure S5b shows the instantaneous frequency of the typical heterodyne waveform and the corresponding terahertz frequency derived from the Hilbert transform. It has as large frequency modulation as the bandwidth of the comb spectrum in Figure 1c.

Note 6 **Feedback amplitude necessary for passive mode-locking**

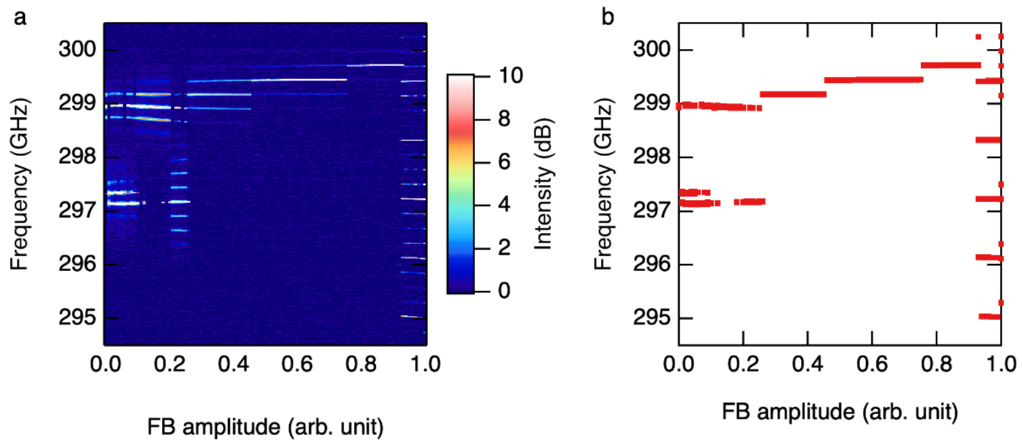

Fig. S6 Change of the spectrum measured when the feedback amplitude was decreased from the passively mode-locked state. **a**, Color plot of the spectrum. **b**, Frequency of the peaks with the intensity larger than 20 dB in **a**. The horizontal axis is the relative feedback field amplitude in our setup.

Figure S6 shows the change of the spectrum when the feedback from the mirror was decreased from the passively mode-locked state. The passive mode-locked state is represented by the equidistant peaks observed when the feedback amplitude is close to its maximum value in our setup. It disappeared when the feedback amplitude was decreased to less than 93 %.

Note 7 Detail of the hybrid mode locking

(1) Comparison of the passive and hybrid mode-locked spectra

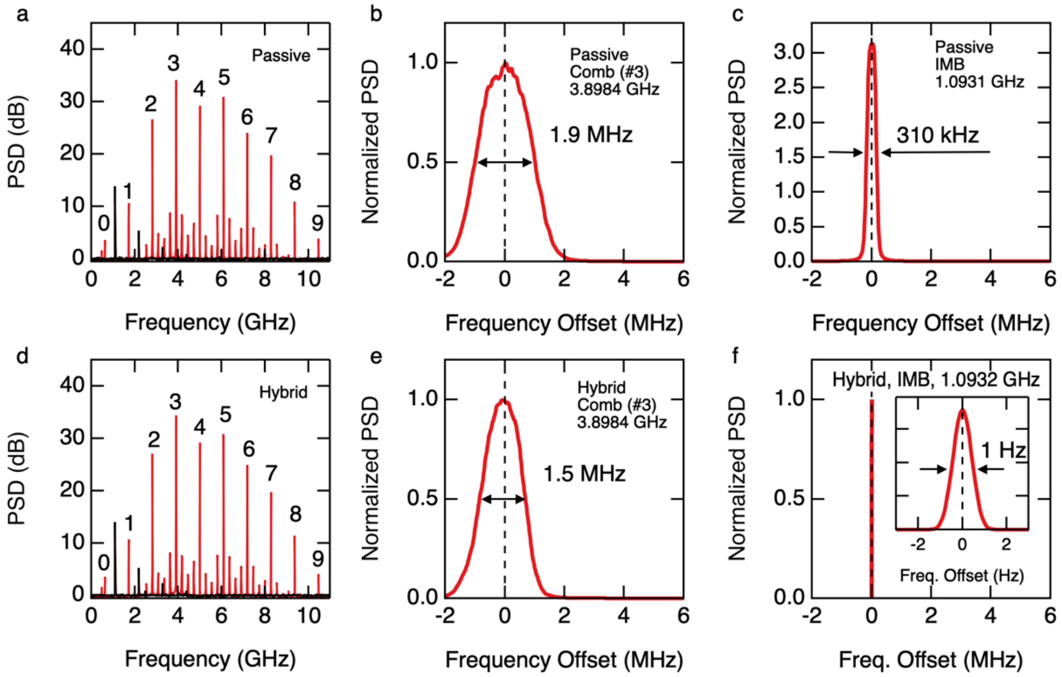

Fig. S7-1 RF spectrum in the passively mode-locked state and the hybrid mode-locked state with modulation of -40 dBm. **a**, Entire heterodyne spectrum (red trace) and the inter-mode beat note (black trace) in the passively mode-locked state. **b**, Magnified view of the comb line indexed as 3. **c**, Magnified spectrum of the inter-mode beat note. **d-f**, Corresponding spectra to a-c in the hybrid mode-locked state.

Figure S7-1 compares the RF spectrum in the passively mode-locked state and the hybrid mode-locked state when the output power of the modulator is -40 dBm. Figures S7-1a and d show the amplitudes of the comb lines. They do not change due to the modulation. Figures S7-1b and e show the linewidths of the comb lines. They also do not change largely by the hybrid mode-locking. It means that the carrier-envelope-offset frequency is not stabilized by the modulation. Figures S7-1c and f show the linewidths of the inter-mode beat notes. It becomes much smaller by the hybrid mode-locking. For two reasons, this signal can be identified as the homodyne signal of the terahertz emission instead of an RF signal directly from the RF modulator. First, it disappeared merely by blocking the terahertz emission from the RTD oscillator. Second, as shown in Fig. S7-2c, its amplitude did not depend on the modulation amplitude below -30 dBm and even decreased with -20 dBm modulation.

(2) Conditions for hybrid mode-locking

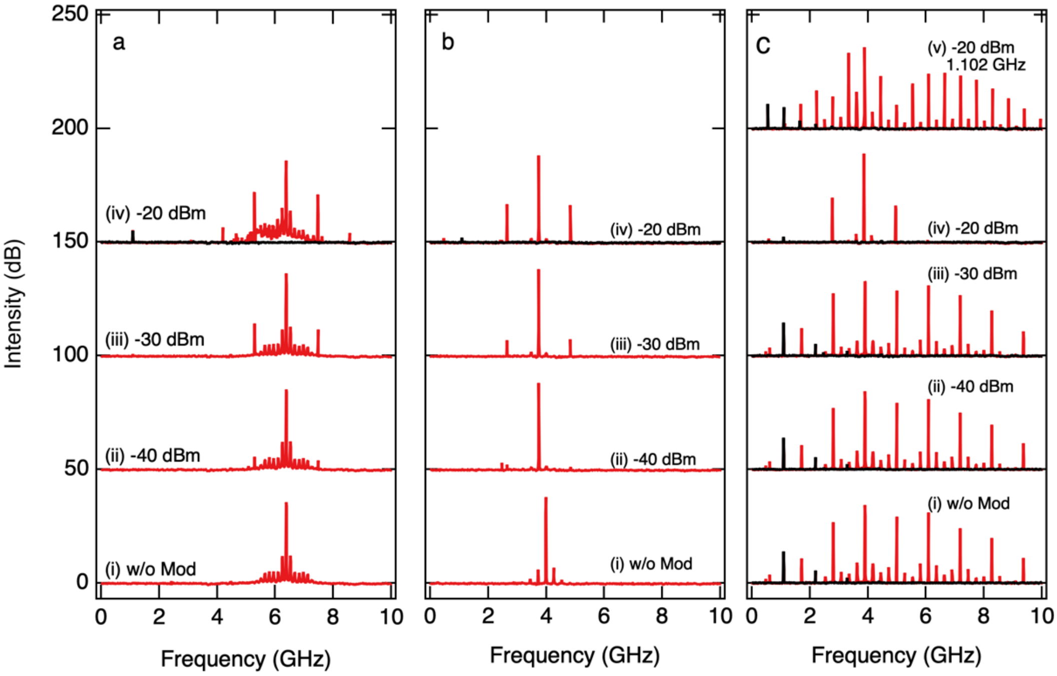

Fig. S7-2 Heterodyne spectra (red) and homodyne spectra (black) for various conditions of the return light and the modulation power. **a,** Return light was blocked. **b**, Return light was injected, but the mirror position was not suitable for the passive mode-locking. **c**, Return light was injected, and the passive mode-locking took place. For each case, the modulation powers were (i) 0 (no modulation), (ii) -40 dBm, (iii) -30 dBm, and (iv) -40 dBm. These spectra are offset by 50 dBm. The modulation frequency was 1.0932 GHz, which is the harmonic-comb spacing in **c** (i). **c** (v) corresponds to the modulation power of -20 dBm and the modulation frequency of 1.1023 GHz.

Here, we discuss the conditions of the optical feedback and the modulation power to obtain the hybrid mode-locked state. We show that the passive mode-locking mechanism is necessary to obtain a broadband comb spectrum.

Figures S7-2a, b, and c show the spectra measured in different optical-feedback conditions. Figure S7-2a shows the spectra measured without feedback from the mirror. Figure S7-2b shows the spectra measured when the feedback with the feedback from the mirror, but its position was not suitable for the passive mode-locking. In these cases, a comb spectrum was not obtained for any modulation power.

When the feedback condition is suitable for the passive mode-locking, a small bias-voltage modulation was efficient to stabilize the comb. Figure S7-2c shows the spectra under various modulation conditions. Figure S7-2c (i) shows the passively mode-locked spectrum observed without the modulation. Figure S7-2c (ii) and (iii) show the spectra under the bias-voltage modulation of -40

dBm and -30 dBm, respectively. The modulation frequency was 1.0932 GHz, which was the same as the harmonic-comb spacing of the passively mode-locked state.

When the modulation amplitude is further increased, the condition to obtain the mode-locked state becomes complicated. The amplitudes of the modes did not change largely, and the hybrid mode-locking was achieved. Figure S7-2c (iv) shows the spectrum under a modulation with a power of -20 dBm and a frequency of 1.0932 GHz. In this case, a comb spectrum was not obtained. Figure S7-2c (v) shows the spectrum under a modulation with a power of -20 dBm and a frequency of 1.1023 GHz. In this case, a comb spectrum was obtained. It is a remained task to reveal the range of modulation frequency and amplitude where we can obtain the harmonic mode-locking.

Finally, we note that we investigated only a limited part of the vast parameter space, such as modulation frequencies, amplitude, and feedback parameters. Investigating such parameter space would be an important future task to understand the hybrid mode-locking mechanism.

Note 8 **Circuit simulation**

(1) Circuit geometry and parameters

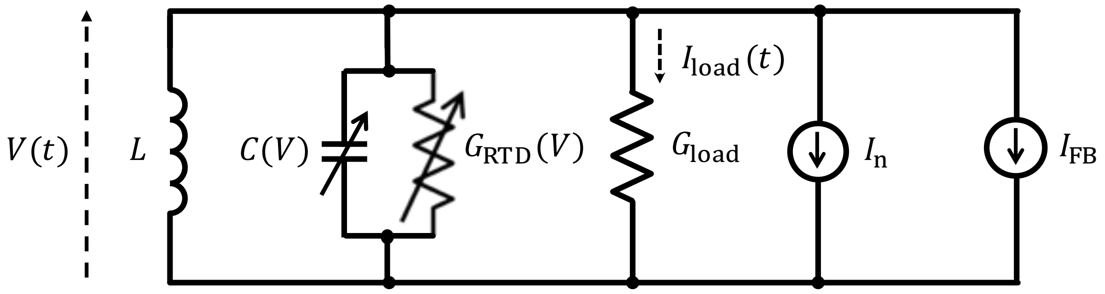

Fig. S8-1 Circuit diagram used in the simulation.

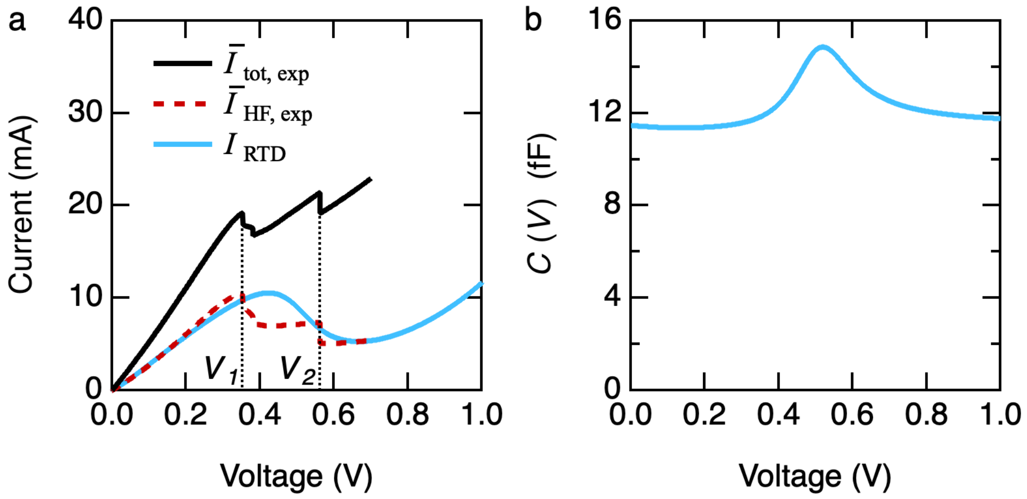

Fig. S8-2 Nonlinear current-voltage curve $I_{\mathrm{RTD}}(V)$ and nonlinear capacitance $C(V)$ used in the simulation. **a**, Current-voltage curve. I_{RTD} is the current-voltage curve of the RTD used in the simulation. As shown in Figure S8-2a, the device consists of high-frequency and low-frequency circuit. $\bar{I}_{\mathrm{tot,exp}}$ is the experimentally measured time-averaged current in the entire circuit. $\bar{I}_{\mathrm{HF,exp}}$ is the time-averaged current in the high-frequency circuit estimated from $\bar{I}_{\mathrm{tot,exp}}$. Experimentally, oscillation took place between the bias voltage of V_1 and V_2, indicated by the dotted vertical lines. **b**, Nonlinear capacitance.

We modeled the RTD oscillator under optical feedback with a circuit diagram of Fig. S8-1. The circuit is the LCR parallel oscillator with a nonlinear conductance $G_{\mathrm{RTD}}(V) = I_{\mathrm{RTD}}(V)/V$ and a nonlinear capacitance $C(V)$ of RTD. For $I_{\mathrm{RTD}}(V)$, the following function [3][4] was used:

$$I_{\mathrm{RTD}}(V) = C_1 V^i \{\arctan C_2(V - V_{\mathrm{th}}) - \arctan C_2(V - V_{\mathrm{n1}})\} + C_3 V^j. \tag{8}$$

For $C(V)$, we used the following function:

$$C(V) = -C_5 V^k \left(\frac{C_6}{1 + {C_6}^2 (V - V_{\text{th}})^2} - \frac{C_6}{1 + {C_6}^2 (V - V_{\text{n2}})^2} \right) + C_4. \quad (9)$$

Equation (9) is our original function based on the previous studies showing that the nonlinear capacitance of the RTD is proportional to $I_{\text{RTD}}(V)$ [5] [6] [7]. Equation (9) is obtained by taking the derivative of equation (8) and picking the major terms that contribute to the nonlinearity around the inflection point of $I_{\text{RTD}}(V)$.

The parameters of equations (8) and (9) are shown in Table S8-1. Figure S8-2 is the plot of $I_{\text{RTD}}(V)$ and $C(V)$. The parameters of $I_{\text{RTD}}(V)$ were decided from the experimentally measured current-voltage curve, as described in Supplementary Note 8 (2). The nonlinear capacitance $C(V)$ was tuned so that the simulation reproduces the frequency-voltage curve of the oscillator, as shown in Figures 3, 4, and S8-4.

The passive elements of the circuit were $L = 21.2$ pH and $G_{load} = 3.3$ mS. I_{n} is a white noise source. Shot noise has the root-mean-square current fluctuations of 162 μA and the single-sided bandwidth of 10 THz. We did not include the shot-noise enhancement of the RTD [8].

The optical feedback was modeled as the feedback current

$$I_{\text{FB}} = \sqrt{\eta} I_{\text{load}}(t - t_d). \quad (10)$$

Here, η is the *reflectivity* which includes the coupling efficiency, and t_d is the time delay. When we include several return lights from several surfaces, the contributions from these return lights were included as a summation:

$$I_{\text{FB}} = \sum_n \sqrt{\eta_n} I_{\text{load}}(t - t_{d,n}). \quad (11)$$

Here, η_n and $t_{d,n}$ are reflectivities and time delays for each return light. The included feedback parameters are shown in Supplementary Note 8 (3).

The simulation was performed using LTspice.

Table S8-1 Parameters of $I_{\text{RTD}}(V), C(V)$

Function	Parameter	Value	Unit
$I_{\text{RTD}}(V)$	V_{th}	0	V
	V_{n1}	0.5068	V
	C_1	0.01025	$\text{A} \cdot \text{V}^{-i}$
	C_2	11.43	V^{-1}
	C_3	0.01071	$\text{A} \cdot \text{V}^{-j}$
	i	0.9107	-
	j	3.440	-
$C(V)$	V_{th}	0	V
	V_{n2}	0.5031	V

$C(V)$	C_4	11.453	fF
	C_5	1.109	$\mathrm{fF \cdot V^{1-k}}$
	C_6	10.40	$\mathrm{V^{-1}}$
	k	1.765	-

(2) Determination of current-voltage curve

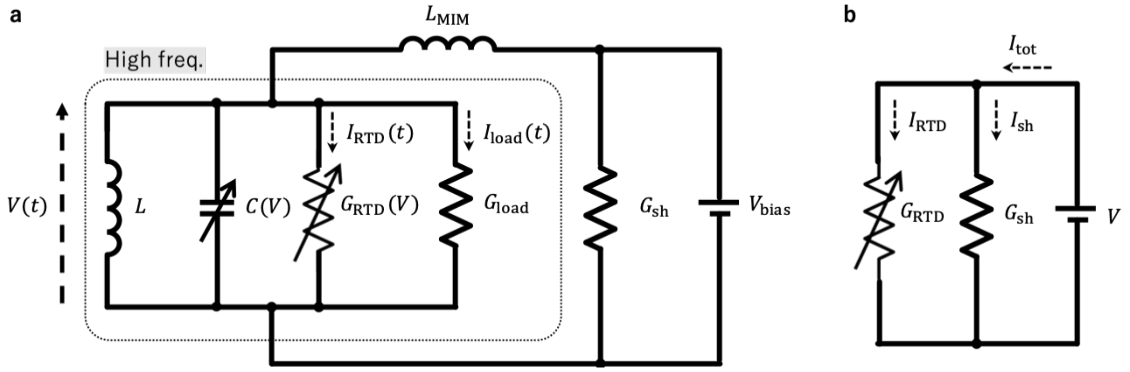

Fig. S8-3 Schematic circuit diagram of the oscillator including the low-frequency circuit. **a**, Entire circuit. **b**, Reduced circuit for the non-oscillating case.

Here, we show how we determined $I_{\mathrm{RTD}}(V)$ used for the simulation.

Figure S8-3a is a schematic diagram of the oscillator including the low-frequency circuit, which was not shown in Figure S8-1. The circuit boxed with the dotted line is the high-frequency circuit that oscillates in the terahertz frequency. The remaining part is the low-frequency circuit consists of a DC voltage source V_{bias}, a shunt conductor G_{sh}, and a MIM capacitor represented as an inductance L_{MIM}. When the circuit is not oscillating, the circuit can be simplified to Figure S8-3b by neglecting the inductance and conductance of the antenna L and G_{load}, capacitance $C(V)$, and the inductance L_{MIM}.

We defined $I_{\mathrm{RTD}}(V)$ so that the following condition is satisfied: $I_{\mathrm{RTD}}(V)$ and $I_{\mathrm{HF,exp}}(V)$, the current in the high-frequency circuit derived from the experiment, show similar curves when the circuit is not oscillating.

We measured $\bar{I}_{\mathrm{tot,exp}}(V)$ shown in Figure S8-2a, the time-averaged current in the entire circuit. $\bar{I}_{\mathrm{HF,exp}}(V)$ is estimated from $\bar{I}_{\mathrm{tot,exp}}(V)$ as follows:

$$\bar{I}_{\mathrm{HF,exp}}(V) = \bar{I}_{\mathrm{tot,exp}}(V) - G_{\mathrm{sh}}V. \tag{12}$$

Here, we assumed $G_{\mathrm{sh}} = 25\ \mathrm{mS}$ to satisfy

$$\frac{d\bar{I}_{\mathrm{HF,exp}}}{dV} \sim 0 \tag{13}$$

when the bias voltage is just outside the range where the oscillation takes place. When the circuit is not oscillating, $I_{\mathrm{HF,exp}}(V)$ is a DC current and the same as the averaged current $\bar{I}_{\mathrm{HF,exp}}(V)$.

$dI_{\mathrm{HF,exp}}/dV$ is the differential conductance of the high-frequency circuit. It should be zero at the edge of the bias-voltage range where the oscillation takes place. The obtained $I_{\mathrm{HF,exp}}(V)$ is shown in Figure S8-2a.

By tuning the parameters, we obtained $I_{\mathrm{RTD}}(V)$ resembles $\bar{I}_{\mathrm{HF,exp}}(V)$ when the circuit is not oscillating.

(3) Determination of feedback delays and reflectivity

In the simulation, we included three return lights shown in Table S8-2. In this section, we describe how we determined the feedback delay and reflectivity of the return lights included in our model.

Table S8-2 Parameters of the return lights included in the simulation. Here, # is the index to identify the return light. The optical length is $l = ct_d/2$, where t_d is the delay time, and c is the speed of the light in the vacuum. The feedback parameter is $C = \sqrt{\eta}\omega_0 t_d/Q$ as introduced in equation (3). In the calculation of C, we assumed f_0=300 GHz and Q=8.

#	Delay t_d (ps)	Optical length l (mm)	Reflectivity η	Feedback parameter C	Expected object	Effect
1	19.7	2.95	$10^{-2.0}$	0.46	Horn antenna	Frequency jump
2	178	26.7	$10^{-3.0}$	1.3	Mount	
3	3340	500	$10^{-3.25}$	19	Mirror	Multiple longitudinal modes

(3-1) Return lights causing the frequency jump (#1 and #2)

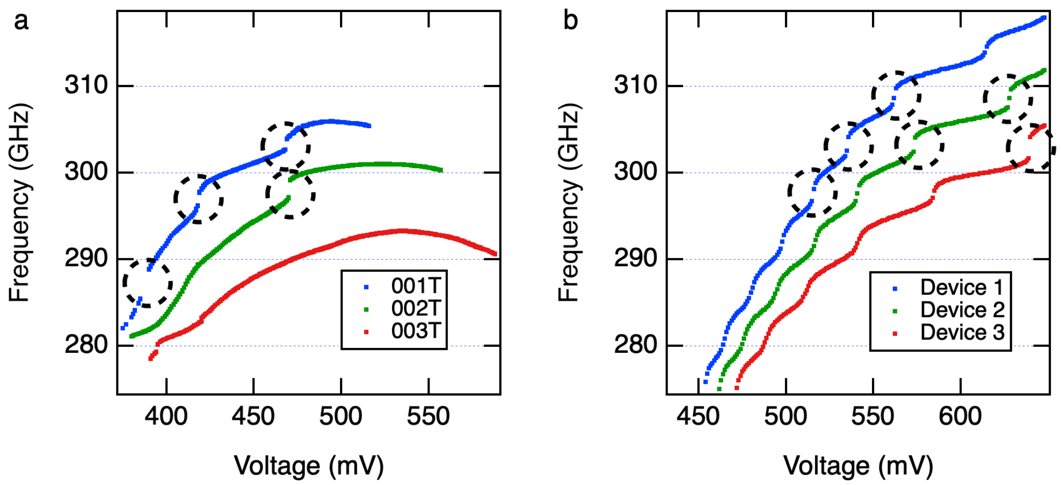

Figure S8-4 Frequency-voltage curves of three oscillators of the same design. **a**, Experimental result for oscillators named 001T, 002T, and 003T. **b**, Simulation result. The inductance L is assumed to be 20.5, 21.2, and 21.9 pH in Device 1, 2, and 3, respectively.

As explained with a simple model in Supplementary Note 5, the frequency jump is originated from the optical feedback from a surface nearby the oscillator. Figure S8-4a shows frequency-voltage curves of three oscillators of the same design but have some variation in actual properties. We found that the number of the frequency jump is different for three oscillators. These characteristics cannot be explained with a single return light because it causes equidistant frequency jumps as expressed by equation (2). Figure S8-4b shows that a simulation reproduced them by assuming two reflection surfaces with the parameter (t_d, η) of $(19.7\,\mathrm{ps}, 10^{-2.0})$ and $(178\,\mathrm{ps}, 10^{-3.0})$. We expect that those reflections are caused at the edge of the horn antenna structure [9] and at an optical mount of the oscillator. The variation of the three oscillators was reproduced by assuming a variation of the inductance L.

Here, we did not correct the detailed discrepancy in the voltage value because we intend to reproduce the behavior of the oscillator qualitatively. We also ignored the increase of the simulated frequency at the high-voltage limit.

(3-2) Return light from the mirror (#3)

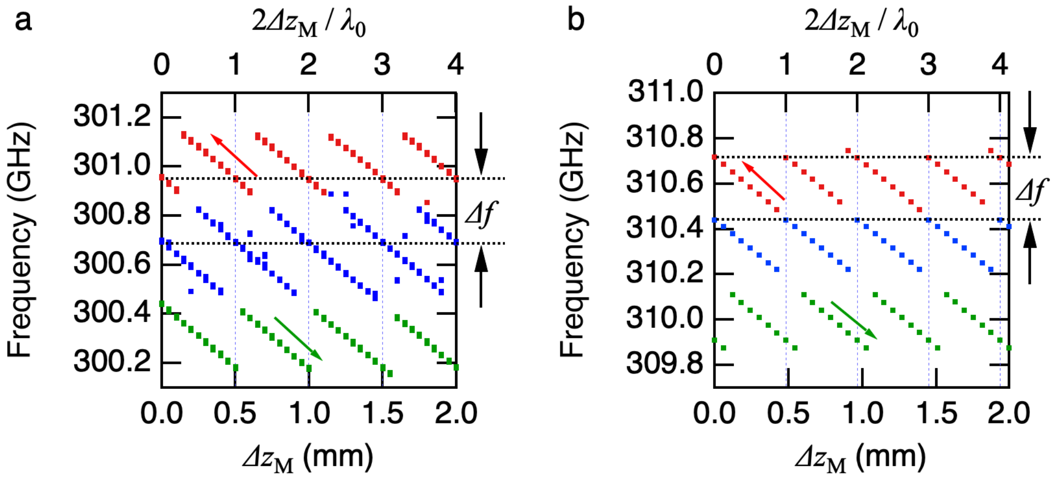

Figure S8-5 Change of the oscillation frequency when the mirror was swept. **a**, Experimental result. The bias voltage was 500 mV, which is expected to be close to the inflection point of the current-voltage curve of the RTD. The red and green dots show the results for the different sweep directions. The blue dots shows the results for the measurement without hysteresis effect, in which an optical chopper is rotating in front of the RTD oscillator. **b**, Simulation result. The bias voltage was 520 mV, which is the inflection point of the current-voltage curve of the RTD. The red and green dots were simulated with sweeping the feedback delay time. The blue dots were simulated without the sweep.

The reflectivity η of the mirror was estimated by the oscillation frequency change observed when the mirror was swept. Figure S8-5a shows the experimentally measured frequency change. The oscillation frequency showed hysteretic behavior depending on the sweep direction, as shown by the green and red dots. The blue dots were measured *without hysteresis effect*, rotating an optical chopper in front of the RTD oscillator. This hysteretic behavior means that the feedback from the feedback parameter C is large, and there are multiple longitudinal modes, as shown in Supplementary Note 5. The mode spacing, shown as Δf in Figure S8-5, was 270 MHz.

As shown in Figure S8-5b, the simulation reproduced the frequency change by the mirror position and the hysteresis. Here, the optical length was swept around 500 mm. The mode spacing changed depending on the reflectivity η. When the reflectivity was $\eta = 10^{-3.25}$, the simulation well reproduced the mode spacing of 270 MHz. The feedback parameter is $C = 19$, which is large enough to cause the multiple longitudinal modes.

(3-3) Confirmation of parameters

Finally, we confirmed that the three return lights estimated above reproduce the observed behavior. The simulated frequency-voltage curve shown in Figure 4a well reproduced the frequency-voltage curve of Figure 3a. Hence, we utilized the parameters of the return lights discussed above.

We note that slight return lights from the lens shown in Figure 1a and the detector were neglected in the simulation. In the experiment, we confirmed that these return lights had a small effect on the oscillator, for example, a change of the linewidth in the CW oscillation state and a slight shift of the frequency-jump voltage. However, the amplitude of these return lights was estimated to be so small that we did not take these return lights in our simulation.

Note 9 Terahertz waveform in simulation

(9-1) Temporal waveform corresponding to Fig. 4b

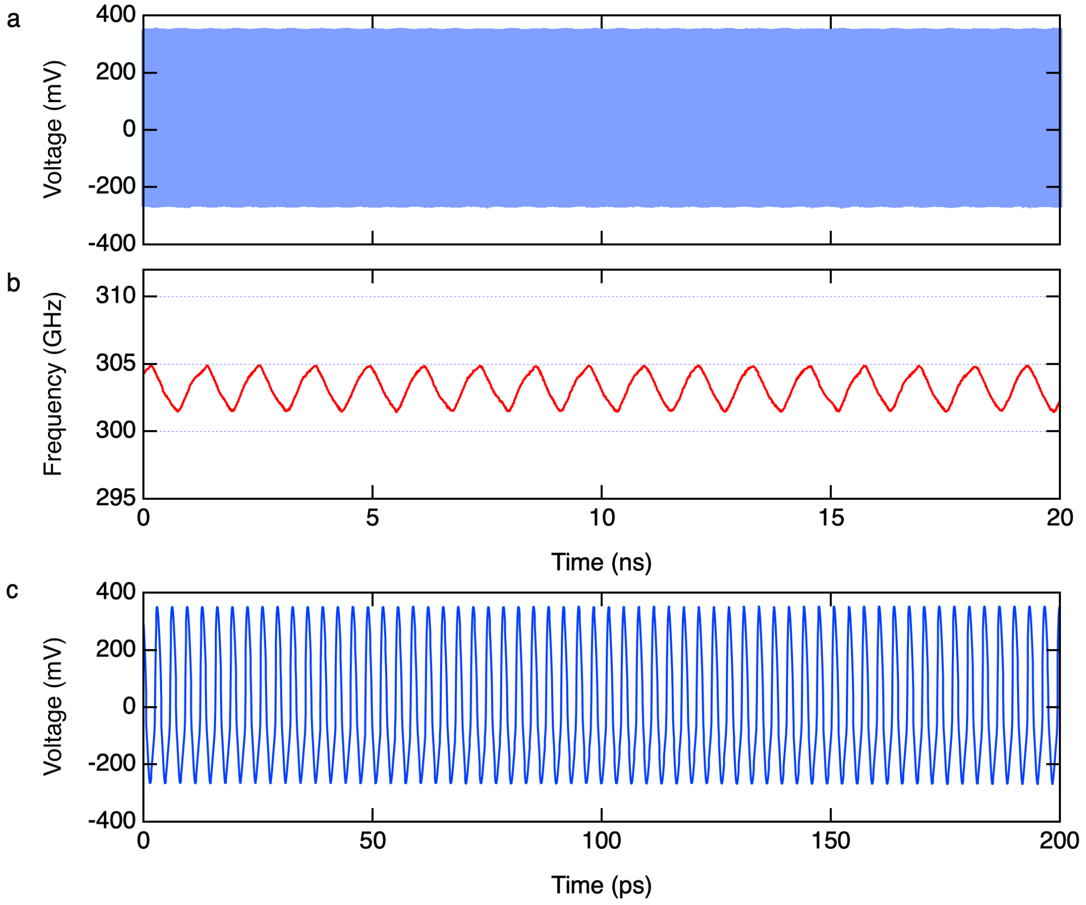

Figure S9-1 Simulated terahertz waveform that corresponds to the harmonic comb spectrum in Figure 4b. The simulation includes noise with a standard deviation of 1/10 of the shot noise. **a,** Waveform for 20 ns (filling the region inside the envelope). **b**, Instantaneous frequency obtained from the terahertz waveform by the Hilbert transform. **c**, Magnified view of the terahertz waveform.

Figure S9-1a shows the simulated terahertz waveform that corresponds to the harmonic frequency comb in Figure 4b. The oscillation frequency is so fast that the waveform is filling the envelope. Clearly, it does not show a significant amplitude modulation. A magnified view of the terahertz waveform is shown in Figure S9-1c.

Figure S9-1b is the instantaneous frequency obtained by the Hilbert transform of the terahertz waveform in the following steps: (i) The simulated waveform has harmonic components such as second harmonics around 600 GHz, third harmonics around 900 GHz, and the other higher harmonics. To obtain the modulation of the fundamental frequency, these harmonic components and low-frequency noise were removed with a band-pass filter of 250 GHz to 350 GHz. (ii) We applied the

Hilbert transform to the filtered temporal waveform. The instantaneous frequency in Figure S9-1b is modulated with a period of approximately 1.2 ns, which is the inverse of the comb spacing of 835 MHz in Figure 4b. The frequency modulation was as large as the spectral bandwidth.

(9-2) Temporal waveform corresponding to Fig. 4c

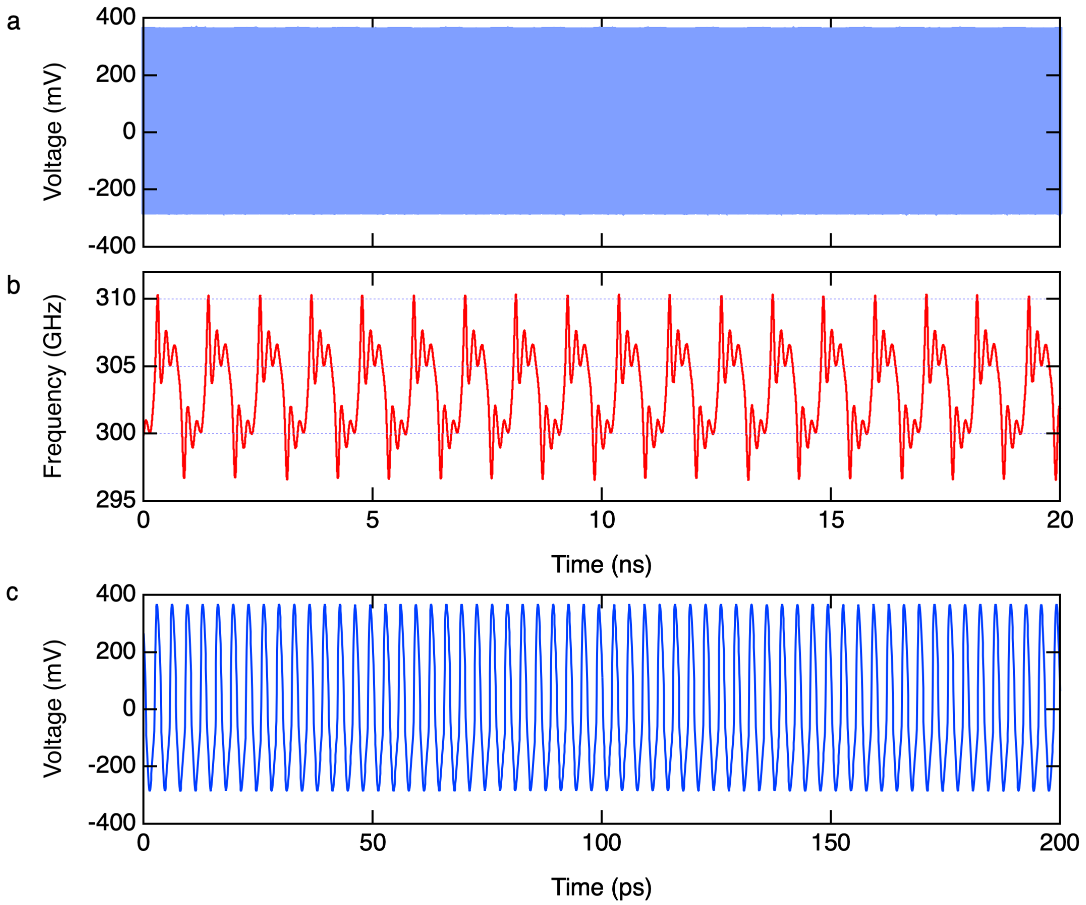

Figure S9-2 Simulated terahertz waveform that corresponds to the harmonic comb spectrum in Figure 4c. The simulation includes noise with a standard deviation of 1/10 of the shot noise. **a,** Waveform for 20 ns (filling the region inside the envelope). **b**, Instantaneous frequency obtained from the terahertz waveform by the Hilbert transform. **c**, Magnified view of the terahertz waveform.

Figure S9-2a shows the simulated terahertz waveform that corresponds to the broadband harmonic frequency comb in Figure 4c. It does not show a significant amplitude modulation. A magnified view of the terahertz waveform is shown in Figure S9-2c. Figure S9-2b is the instantaneous frequency obtained in the same procedure as Figure S9-1b. The frequency modulation is larger than that of Figure S9-1b, reflecting the broader spectrum.

Note 10   Broadband frequency combs

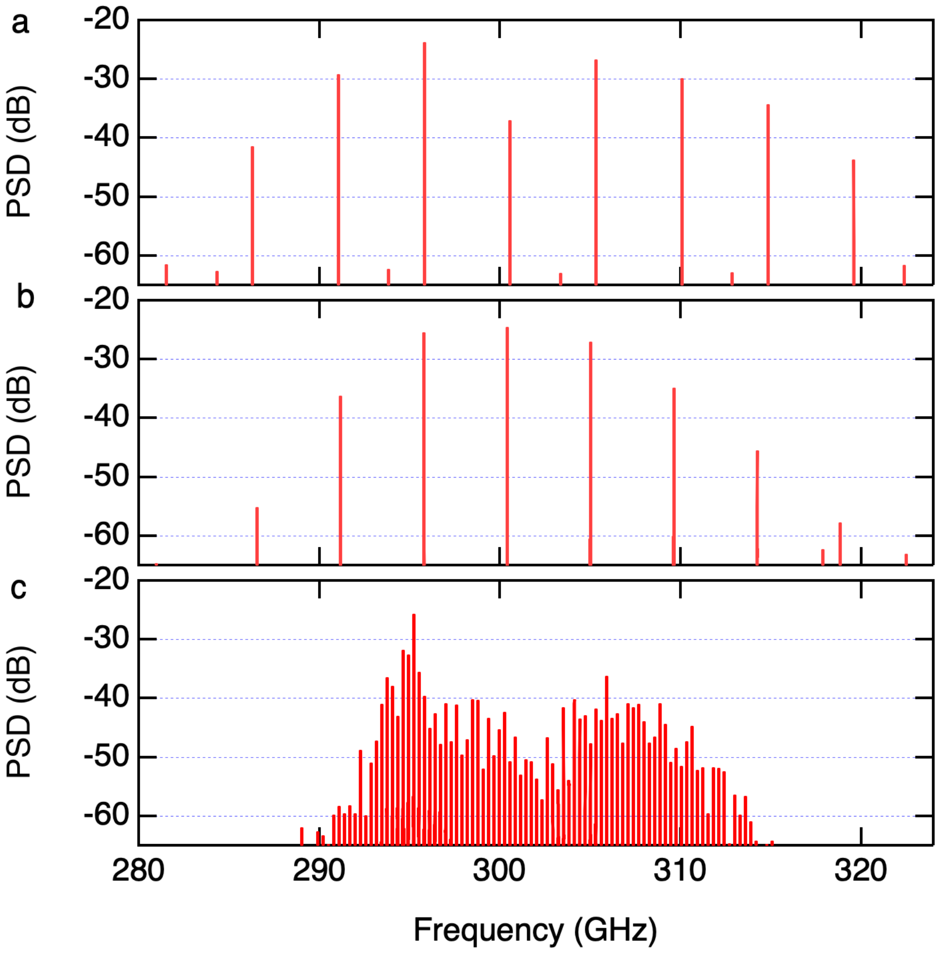

Fig. S10 Broadband comb spectra simulated for the conditions shown in Table S10.

Table S10 Feedback conditions for Figure S10a, b, and c. The reflectivities at the reflection surfaces and their delays are shown. The spacing of the comb lines are also shown.

**Figure**	**Delay and reflectivity**			**Comb spacing (GHz)**
	**#1**	**#2**	**#3**	
	**19.7 ps**	**178 ps**	**3340 ps**	
S10a	10^{-1}	10^{-2}	$10^{-2.25}$	4.76
S10b	10^{-1}	10^{-2}	0	4.61
S10c	10^{-1}	0	10^{-2}	0.296

Figure S10 shows comb spectra simulated for various feedback conditions, and Table S10 shows the conditions. The distances of the reflection surfaces are the same as those in Table S7-2. Depending on the feedback conditions, we obtained various broadband comb spectra.

References

[1] D. Lenstra, M. Van Vaalen, B. Jaskorzyńska, On the theory of a single-mode laser with weak optical feedback. *Physica B+C.* **125**, 255–264 (1984).

[2] M. Asada, S. Suzuki, Theoretical analysis of external feedback effect on oscillation characteristics of resonant-tunneling-diode terahertz oscillators. *Jpn. J. Appl. Phys.* **54**, 070309 (2015).

[3] Brown, E. R., McMahon, O. B., Mahoney, L. J. & Molvar, K. M. SPICE model of the resonant-tunnelling diode. *Electron. Lett.* **32**, 938–940 (1996)

[4] Ternent, G. & Paul, D. J. SPICE modeling of the scaling of resonant tunneling diodes and the effects of sidewall leakage. *IEEE Trans. Electron Devices* **59**, 3555–3560 (2012)

[5] Wei, T. & Stapleton, S. Effect of spacer layers on capacitance of resonant tunneling diodes. *J. Appl. Phys.* **76**, 1287–1290 (1994)

[6] Qingmin Liu, Seabaugh, A., Chahal, P. & Morris, F. J. Unified AC model for the resonant tunneling diode. *IEEE Trans. Electron Devices* **51**, 653–657 (2004)

[7] Asada, M., Suzuki, S. & Kishimoto, N. Resonant Tunneling Diodes for Sub-Terahertz and Terahertz Oscillators. *Jpn. J. Appl. Phys.* vol. 47 4375–4384 (2008)

[8] Asada, M. Theoretical analysis of spectral linewidth of terahertz oscillators using resonant tunneling diodes and their coupled arrays. *J. Appl. Phys.* **108**, 034504 (2010)

[9] Tsuruda, K. *et al.* Development of Practical Terahertz Packages for Resonant Tunneling Diode Oscillators and Detectors. in *2020 IEEE International Symposium on Radio-Frequency Integration Technology (RFIT)* 193–195 (ieeexplore.ieee.org, 2020).
